# Spatially resolved anomalous dispersion method for determination of site-specific oxidation states in metalloenzymes at XFEL sources

**DOI:** 10.1063/4.0001025

**Published:** 2025-10-27

**Authors:** Natalie M Minnetian, Nicholas K Sauter, Jan F Kern

**Affiliations:** Lawrence Berkeley National Laboratory, Berkeley, CA, USA

## Abstract

Catalysis by metalloenzymes is controlled by precise movement of protons and electrons at active sites which are often coordinated with ligand binding, side-chain movement, or larger protein conformational changes. We seek to understand the mechanism of Photosystem II (PSII), where the coordinated movement of substrate and electrons within a Mn_4_CaO_5_ cofactor catalyzes biological water oxidation. To fully characterize mechanisms of PSII and other enzymes, simultaneous interrogation of protein structure and oxidation state of each metal cofactor is required. These research questions can be addressed by X-ray crystallography by analyzing the anomalous scattering of each metal atom, even for metallocofactors which contain multiple copies of the same metal type. The spatially resolved anomalous dispersion (SPREAD) method depends on the wavelength dependent behavior of scatterers around their absorption K-edge. Time-resolved crystallographic methods using X-ray free electron lasers (XFELs) could provide spatial and temporal resolution of oxidation states, but adapting SPREAD for use with XFELs is inherently challenging due to the shot-to-shot variability and stochastic nature of the XFEL pulses. We present progress towards extending the SPREAD method to XFELs, taking advantage of the pulse distribution to collect entire datasets without the need for monochromatic X-rays, and discuss methods for data processing. With one data analysis method, energy contributions to each pixel contributing to Bragg spots are computationally modeled, allowing structure factors to be extracted. As an alternative to the pixel-based approach, we also explore assignment of energies to whole spots as a means to determine energy dependent scattering factors. Developing the software for time-resolved SPREAD experiments enables serial crystallography experiments to isolate transient intermediates that are important in catalysis, including the four metastable intermediates in the catalytic cycle of PSII, and the elusive S4 state where O-O bond formation occurs.

